# Editorial: Automations in Long-Term Neurorehabilitation

**DOI:** 10.3389/fneur.2022.864953

**Published:** 2022-04-08

**Authors:** Xiaoling Hu, Ping Zhou, Jun Yao, Rong Song

**Affiliations:** ^1^Department of Biomedical Engineering, Hong Kong Polytechnic University, Kowloon, Hong Kong SAR, China; ^2^Faculty of Biomedical and Rehabilitation Engineering, University of Health and Rehabilitation Sciences, Qingdao, China; ^3^Physical Therapy and Human Movement Sciences Department, Northwestern University, Chicago, IL, United States; ^4^School of Biomedical Engineering, Sun Yat-sen University, Guangzhou, China

**Keywords:** automation, neurorehabiliation, rehabilitation service, robotics, artificial intelligence

Neurological disorders, such as stroke, Parkinson's disease, dementia, etc., are increasing globally mainly because of the rapid growth of the aging population and the trend of these diseases in younger generations. Long-term neurorehabilitation for these disorders has been challenging in traditional rehabilitation services, which heavily rely on manual operations by professional manpower in diagnosis, treatment, and follow-up, when the patient populations are expanding. Furthermore, the COVID-19 pandemic has already lasted for years globally with an unforeseeable end-date, which has triggered the urgent need of more flexible rehabilitation, e.g., remote and self-help modes at home, to augment the traditional, centralized, and face-to-face practices in hospitals/clinics. Automations that facilitate self-help management in rehabilitation with minimized close contact and involvement of human professionals, meanwhile without sacrificing the rehabilitation quality, will be a new trend in neurorehabilitation. Its prospect and wide application depend on the advances in automated technologies in diagnosis, selection, and administration of suitable treatment, and longitudinal follow-up. In this Research Topic, we collected six articles that addressed the technologies, or methods, which have great potential for long-term automated neurorehabilitation.

Regular and persistent exercises are recommended for everyone, particularly older people and disabled persons, to maintain body functions for quality life. In the study, “*Tai Chi and Yoga for Improving Balance on One Leg: A Neuroimaging and Biomechanics Study*” by Chen et al., two different popular exercises, Tai Chi and Yoga, were compared in their effectiveness in improving one-leg stance balance. The quantitative cortical activation and moving trajectories during the practices were compared to investigate the possible neurological mechanisms. The study pointed out that Tai Chi could provide more cognitive training that contributed to better postural control than yoga, which may benefit balance in rehabilitation. In the study, “*Detraining Slows and Maintenance Training Over 6 Years Halts Parkinsonian Symptoms-Progression*” by Hortobágyi et al., it was demonstrated that a short-term and agility exercise program could relieve symptoms of Parkinson's disease up to a year during detraining, but the subsequent 6-year maintenance program of less intensity could further increase or sustain the initial improvements in symptoms and quality of life with a minimum drug dose.

Automated diagnosis requires quantitative methods for measurements. The study, “*Quantifying the Changes of Mechanical and Electrical Properties of Paralyzed Muscle in Survivors With Cervical Spinal Cord Injury*” by Hu et al., proposed an evaluation by myotonometry and electrical impedance myography (EIM) with quantified features, compared to the traditional clinical scores, e.g., the Manual Muscle Testing and Modified Ashworth Scale. The results supported the feasibility of using myotonometry and EIM for evaluation of muscle properties in persons with spinal cord injuries. In another study, “*Automated Movement Assessment in Stroke Rehabilitation*” by Ahmed et al., a home-based semi-automated assessment system was introduced for long-term rehabilitation. The system was designed with low-cost and unobtrusive sensors to capture the upper limb movement of a person after stroke via cyber-human methodology; automated assessments on the movement quality could be provided based on an artificial intelligent expert model.

Intelligent automation for rehabilitative intervention can provide a helping hand in labor-demanding long-term service. Kubota et al. introduced a robotic system and its clinical application in the study, “*Robotic Shoulder Rehabilitation With the Hybrid Assistive Limb in a Patient With Delay Recovery After Postoperative C5 Palsy: A Case Report.”* The complete shoulder functions of the patient were restored after 23 sessions of the robot-assisted training. In the study, “*Real-Time Detection of Freezing Motions in Parkinson's Patients for Adaptive Gait Phase Synchronous Cueing*” by Dvorani et al., a state-automaton-based detection of the Freezing of Gait (FoG) sensing method based on machine learning was developed for accurate detection and prediction of specific gait patterns, which can be utilized for closed-loop systems providing on-demand gait phase-synchronous cueing to mitigate FoG systems and to prevent motoric blockades.

We hope that this Research Topic will help to promote further innovations in automated technologies for neurorehabilitation services, not only reducing suffering and improving the quality of life of patients, but also facilitating more efficient and effective management by healthcare providers in the industry.

## Author Contributions

XH drafted the editorial. PZ, JY, and RS revised the manuscript. All authors contributed to the article and approved the submitted version.

## Conflict of Interest

The authors declare that the research was conducted in the absence of any commercial or financial relationships that could be construed as a potential conflict of interest.

## Publisher's Note

All claims expressed in this article are solely those of the authors and do not necessarily represent those of their affiliated organizations, or those of the publisher, the editors and the reviewers. Any product that may be evaluated in this article, or claim that may be made by its manufacturer, is not guaranteed or endorsed by the publisher.

